# Gold Nanoparticles as Boron Carriers for Boron Neutron Capture Therapy: Synthesis, Radiolabelling and In Vivo Evaluation

**DOI:** 10.3390/molecules24193609

**Published:** 2019-10-07

**Authors:** Krishna R. Pulagam, Kiran B. Gona, Vanessa Gómez-Vallejo, Jan Meijer, Carolin Zilberfain, Irina Estrela-Lopis, Zuriñe Baz, Unai Cossío, Jordi Llop

**Affiliations:** 1 Radiochemistry and Nuclear Imaging Group, CIC biomaGUNE, 20014 San Sebastian, Spain; krpulagam@cicbiomagune.es (K.R.P.); kirangonas@gmail.com (K.B.G.); zbaz@cicbiomagune.es (Z.B.); 2 Cardiovascular Molecular Imaging Laboratory, Section of Cardiovascular Medicine and Yale Cardiovascular Research Center, Yale University School of Medicine, New Haven, CT 06511, USA; 3 Veterans Affairs Connecticut Healthcare System, West Haven, CT 06516, USA; 4 Radiochemistry Platform, CIC biomaGUNE, 20014 San Sebastian, Spain; vgomez@cicbiomagune.es; 5 Institute of Medical Physics and Biophysics, Leipzig University, Härtelstrasse 16-18, 04107 Leipzig, Germany; jan.meijer@uni-leipzig.de; 6 Felix Bloch Institute for Solid State Physics, Leipzig University, Linnéstraße 5, 04103 Leipzig, Germany; Carolin.Merker@medizin.uni-leipzig.de (C.Z.); Irina.Estrela-Lopis@medizin.uni-leipzig.de (I.E.-L.); 7 Radioimaging and Image Analysis Platform, CIC biomaGUNE, 20014 San Sebastian, Spain; ucossio@cicbiomagune.es; 8 Centro de Investigación Biomédica en red Enfermedades Respiratorias–CIBERES, 28029 Madrid, Spain

**Keywords:** boron neutron capture therapy, gold nanoparticles, cobalt *bis*(dicarbollide), positron emission tomography, radiolabelling, iodine-124, HT1080

## Abstract

*Background*: Boron Neutron Capture Therapy (BNCT) is a binary approach to cancer therapy that requires accumulation of boron atoms preferentially in tumour cells. This can be achieved by using nanoparticles as boron carriers and taking advantage of the enhanced permeability and retention (EPR) effect. Here, we present the preparation and characterization of size and shape-tuned gold NPs (AuNPs) stabilised with polyethylene glycol (PEG) and functionalized with the boron-rich anion cobalt *bis*(dicarbollide), commonly known as COSAN. The resulting NPs were radiolabelled with ^124^I both at the core and the shell, and were evaluated in vivo in a mouse model of human fibrosarcoma (HT1080 cells) using positron emission tomography (PET). *Methods*: The thiolated COSAN derivatives for subsequent attachment to the gold surface were synthesized by reaction of COSAN with tetrahydropyran (THP) followed by ring opening using potassium thioacetate (KSAc). Iodination on one of the boron atoms of the cluster was also carried out to enable subsequent radiolabelling of the boron cage. AuNPs grafted with mPEG-SH (5 Kda) and thiolated COSAN were prepared by ligand displacement. Radiolabelling was carried out both at the shell (isotopic exchange) and at the core (anionic absorption) of the NPs using ^124^I to enable PET imaging. *Results*: Stable gold nanoparticles simultaneously functionalised with PEG and COSAN (PEG-AuNPs@[**4**]^−^) with hydrodynamic diameter of 37.8 ± 0.5 nm, core diameter of 19.2 ± 1.4 nm and ξ-potential of −18.0 ± 0.7 mV were obtained. The presence of the COSAN on the surface of the NPs was confirmed by Raman Spectroscopy and UV-Vis spectrophotometry. PEG-AuNPs@[**4**]^−^ could be efficiently labelled with ^124^I both at the core and the shell. Biodistribution studies in a xenograft mouse model of human fibrosarcoma showed major accumulation in liver, lungs and spleen, and poor accumulation in the tumour. The dual labelling approach confirmed the in vivo stability of the PEG-AuNPs@[**4**]^−^. *Conclusions*: PEG stabilized, COSAN-functionalised AuNPs could be synthesized, radiolabelled and evaluated in vivo using PET. The low tumour accumulation in the animal model assayed points to the need of tuning the size and geometry of the gold core for future studies.

## 1. Introduction

Despite recent advances, an estimated 14.1 million new cancer cases and 8.2 million cancer-related deaths occurred in 2012 [1]. The International Agency for Research on Cancer (IARC) predicts a substantive increase to 19.3 million new cancer cases per year by 2025, due to growth and ageing of the global population [1]. Hence, there is an urgent need to develop new diagnostic and therapeutic tools. 

Binary approaches to cancer therapy, in which two low-toxicity modalities are co-localised in the tumour to become active, are a promising strategy to improve patient outcomes and decrease side effects. Two examples are photodynamic therapy (PDT) [2,3] and photothermal therapy (PTT) [4,5], which are established techniques in which photosensitising agents are administered, followed by targeted irradiation of the treated area with visible or near-infrared (IR) laser light. However, PDT and PTT are limited by the low penetration of visible and IR light into tissues, which limits their application to superficial skin tumours or those at the surface of internal organs or cavities. 

Another binary approach to cancer therapy is Boron Neutron Capture Therapy (BNCT), which is based on the ability of the stable isotope boron-10 (^10^B) to capture thermal neutrons. The interaction of an incident thermal neutron with a ^10^B atom results in the ^10^B(n, α, γ)^7^Li nuclear reaction [6]. Α Particles and ^7^Li recoil ions have high linear energy transfer properties and path lengths in the range of the diameter of a single cell. Hence, if cancer cells selectively take up a sufficient amount of ^10^B and are irradiated with thermal neutrons (ca. 0.5–10 keV), the α particles and ^7^Li ions produced as a consequence of the ^10^B(n, α, γ)^7^Li nuclear reaction result in cellular damage and trigger cell death, while sparing healthy surrounding tissue. 

Over years, abnormal metabolism and the over-expression of membrane receptors have been widely exploited to develop drug candidates capable to accumulate ^10^B nuclei in cancer cells. In this context, boronated carbohydrates, amino acids, peptides, nucleic acids and immunoconjugates [7,8] have been proposed as potential BNCT drug candidates. Unfortunately, only two compounds are currently applied in the clinical practice, i.e., sodium borocaptate (BSH) and *p*-boronophenylalanine (BPA) [9], and their application is restricted to a limited number of tumour types [10].

In the recent years, researchers have exploited the enhanced permeability and retention (EPR) effect to achieve passive accumulation of nanosized drugs in a wide range of tumours. This phenomenon has opened new opportunities for the development of drug-enriched nanomedicines with potential application in BNCT. To date, nanocarriers such as liposomes [11,12,13,14,15], carbon [16] and boron nitride nanotubes [17,18,19,20], magnetic nanoparticles [21,22], boron carbide nanoparticles [23,24] and borosilicates [25] have been synthesized and eventually investigated as BNCT drugs at the pre-clinical level. 

Among all nanomaterials, multifunctional gold nanoparticles (AuNPs), composed of an inorganic metallic gold core surrounded by an organic and/or biomolecular monolayer, have become especially relevant in biomedicine. The core material is chemically inert and non-toxic, both shape and size can be easily tuned and monolayer properties can be modulated due to the well-defined surface chemistry of gold [26]. Consequently, different functionalized gold assemblies have been synthesized and evaluated in different biomedical applications [27,28,29], including their use as potential boron carriers in the context of BNCT. In one of the first works, Schmid et al. reported on the preparation of gold clusters functionalised with BSH using a phase transfer reaction over 6 weeks [30]. Subsequent works comprise the preparation of polymer-stabilized gold nanoparticles using *o*-carborane functionalised polystyrene [31], the preparation of mercaptocarborane- [32,33], *o*-carborane- [34,35] and borane-capped AuNPs [36], and the preparation of carborane-capped gold nanoclusters [37]. 

Here, we present the preparation of size and shape-tuned gold NPs functionalized simultaneously with poly(ethylene glycol) methyl ether thiol and the inorganic, boron-based molecule cobalt *bis*(dicarbollide), [3,3′-Co(1,2-C_2_B_9_H_11_)_2_]^−^, commonly known as COSAN, a stable and water soluble complex in which the cobalt atom is sandwiched between two η^5^-bonding [C_2_B_9_H_11_]^2−^ moieties [38,39]. The resulting functionalized NPs were radiolabelled with ^124^I, a positron emitter with a half-life of 4.17 days, using two different strategies; namely, the radiolabel was incorporated either on the surface of the gold core or covalently attached to the COSAN. Whole body imaging studies using Positron Emission Tomography (PET) were carried out in a mouse cancer model, generated by subcutaneous inoculation of HT1080 (human fibrosarcoma) cells, in order to assess (i) the capability of the multi-functionalized AuNPs to passively accumulate in the tumour; and (ii) the in vivo stability of the NPs and their biological fate. 

## 2. Results and Discussion

### 2.1. Synthesis of COSAN Derivatives

**Scheme 1:**
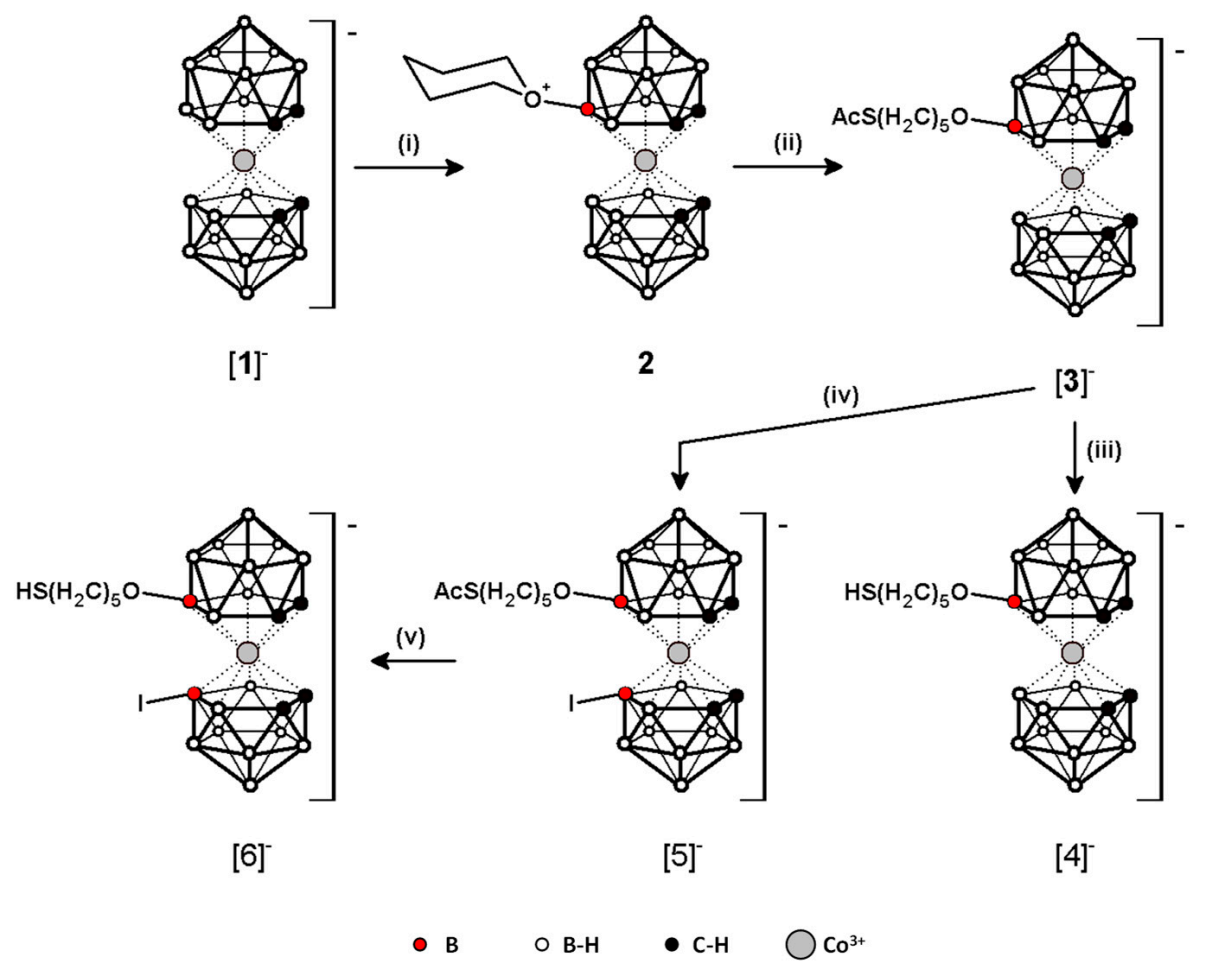
Synthesis of functionalized COSAN derivatives **2**, [**3**]^−^, [**4**]^−^, [**5**]^−^ and [**6**]^−^. (i) THP, dimethylsulphate, H_2_SO_4_; (ii) KSAc; (iii) NaOMe, MeOH; (iv) CH_3_CN, NaI, Chloramine-T; (v) NaOMe, MeOH.

As a first step in the development of the novel boron-rich AuNPs, COSAN derivatives bearing a thiol group for subsequent attachment to AuNPs were synthesized following a previously published methodology [40] with minor modifications (Scheme 1). The reaction of [3,3′-Co(8-C_5_H_10_O-1,2-C_2_B_9_H_10_)(1′,2′-C_2_B_9_H_11_)] (**2**; synthesized as described, [40]) with potassium thioacetate (KSAc) yielded [**3**]^−^ with overall yield of 52%, which led to [**4**]^−^ in almost quantitative yield after basic hydrolysis. In parallel, COSAN derivatives simultaneously incorporating a thiol group and an iodine atom, the latter to enable radiolabelling with the positron emitter ^124^I before attachment to the AuNPs (shell labelling), was achieved by reaction of [**3**]^−^ with sodium iodide, chloramine-T and acetic acid in acetonitrile overnight at room temperature to yield [**5**]^−^ in 69% yield after purification. Basic hydrolysis yielded the thio-derivative [**6**]^−^ ready for incorporation into NPs. Characterization using ^1^H-, ^11^B-, and ^13^C-NMR confirmed the presence of the desired compounds (see experimental section and Supplementary Materials).

### 2.2. Synthesis of Functionalised AuNPs

For successful clinical application, a BNCT drug should deposit a concentration of ^10^B in the tumour in the range 20–35 µg ^10^B/g of tissue, although lower amounts might be sufficient if the boron atoms are internalized into cells [41,42]. Additionally, high tumour-to-normal tissue and tumour-to-blood ratios should be achieved in order to prevent damage to healthy tissue and blood vasculature during neutron irradiation. Achieving these values is highly challenging, and nanotechnology has opened new opportunities due to the well-known EPR effect which results in passive accumulation of nanosized materials in tumour tissue. Here, and based on the previous work reported by Cioran et al. [32] we envisaged the possibility of using gold nanoparticles as boron carriers. Besides the easy functionalization chemistry, gold nanomaterials are chemically inert and non-toxic and both shape and size can be easily tuned [26]. Among all the possibilities, we decided to start with spherical particles with a size within the optimal range to achieve tumour accumulation [43]. In addition to the boron clusters, we decided to incorporate PEG chains on the NP surface in order to enhance the stability of the NPs in biological media and increase circulation time.

**Figure 1:**
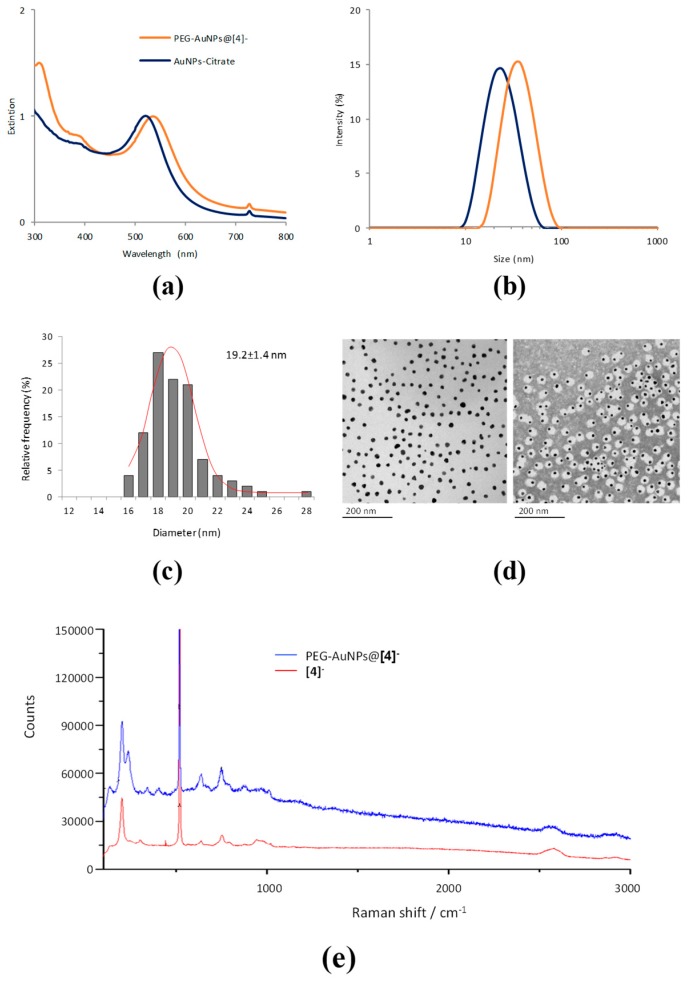
(**a**) UV-Vis spectra corresponding to citrate stabilized AuNPs (AuNPs-Citrate, blue, λ_max_ = 524 nm) and PEG-stabilized AuNPs@[**4**]^−^ (PEG-AuNPs@[**4**]^−^, red, λ_max_ = 531 nm for the gold plasmon; λ_max_ = 310 nm corresponds to compound [**4**]^−^); (**b**) Size distribution ranges as determined by DLS (volume distribution) for AuNPs-Citrate and PEG-AuNPs@[**4**]^−^ (same colour code as in (**a**)); (**c**) Particle size distribution of PEG-AuNPs@[**4**]^−^ as determined by TEM; (**d**) representative TEM images of AuNPs-Citrate and PEG-AuNPs@[**4**]^−^; (**e**) Raman spectra for PEG-AuNPs@[**4**]^−^ and compound [**4**]^−^.

COSAN functionalized-PEG stabilized gold NPs (PEG-AuNPs@[**4**]^−^) were prepared by mixing spherical, citrate-stabilized 18–20 nm gold NPs (AuNPs-citrate, prepared following the Turkevich method [44]) with a mixture of [**4**]^−^ and 5 KDa PEG-thiol in aqueous media. The amount of COSAN in the final PEG-AuNPs@[**4**]^−^, determined by ICP-MS was estimated to be ca. 0.09 mg of boron/mg of gold. The simultaneous incorporation of COSAN and PEG on the surface of the NPs resulted in a bathochromic shift in the longitudinal surface plasmon resonance (SPR) band from 524 nm to 531 nm (Figure 1a). Additionally, another absorption band appeared at ca. 310 nm, close to the maximum absorption of COSAN (314 nm), confirming successful incorporation of the boron cluster on the surface of the AuNPs.

**Figure 2:**
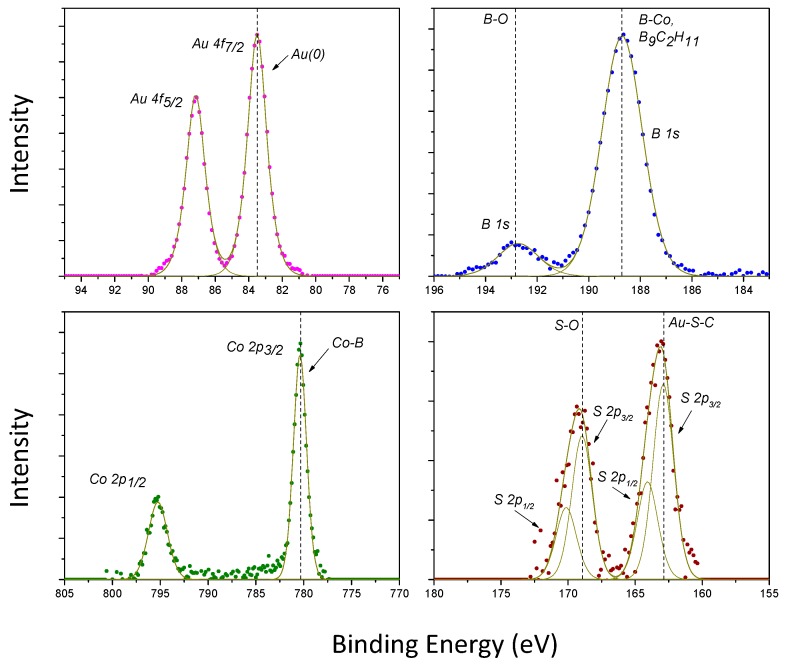
XPS spectra of PEG-AuNPs@[**4**]^−^.

Dynamic light scattering analysis performed on AuNPs-Citrate and PEG-AuNPs@[**4**]^−^ showed a mono-disperse distribution with average hydrodynamic diameter of 24.4 ± 0.5 nm and 37.8 ± 0.5 nm respectively (Figure 1b). TEM analysis revealed the presence of spherical AuNPs with average particle size of 19.2 ± 1.4 nm (Figure 1c,d). PEG-AuNPs@[**4**]^−^ had a negative ξ-potential of −18.0 ± 0.7 mV at neutral pH, as expected after the incorporation of the anionic COSAN complex on the surface, and the Raman spectra showed the presence of absorption bands at 2596−2573 cm^−1^, corresponding to B−H stretching and confirming the presence of the sandwich complex on the NP surface (Figure 1e). Furthermore, X-ray photoelectron spectroscopy (XPS) analysis of PEG-AuNPs@[**4**]^−^ showed a peak at 780.2 eV in the Co 2p spectrum, corresponding to Co-B bonds; peaks at 192.8 and 188.5 eV, corresponding to B-O and C-B bonds in the B 1s spectrum; peaks at 87.5 and 83.5 eV, corresponding to Au 4f5/2 and 4f7/2 respectively; and a peak at 162.5 eV in the S 2p spectrum, corresponding to 2p3/2 Au-S-C bond (Figure 2) [45]. These results confirmed the presence of both cobalt and boron on the surface of AuNPs; furthermore, the NPs have a significant boron load and appropriate size for eventual tumour accumulation.

### 2.3. Radiolabelling of AuNPs

One of the main challenges in BNCT is the determination of the biodistribution of the boron carrier after administration, in order to establish the optimal time window for neutron irradiation. This drawback is even more evident when nanosystems are used as boron carriers, as the determination of the biodistribution and fate of NPs after administration into living systems is extremely challenging. One alternative to overcome this drawback consists of incorporating a positron or gamma emitter into the nanoparticle in order to track the location in a time-resolved fashion using PET or Single Photon Emission Computerised Tomography (SPECT) imaging. This approach has been widely reported in the literature in NPs with very different nature [46,47,48,49,50,51,52]. Additionally, incorporation of the label both at the core and at the shell of NPs can provide very relevant information about the in vivo stability of the NPs [53].

**Figure 3:**
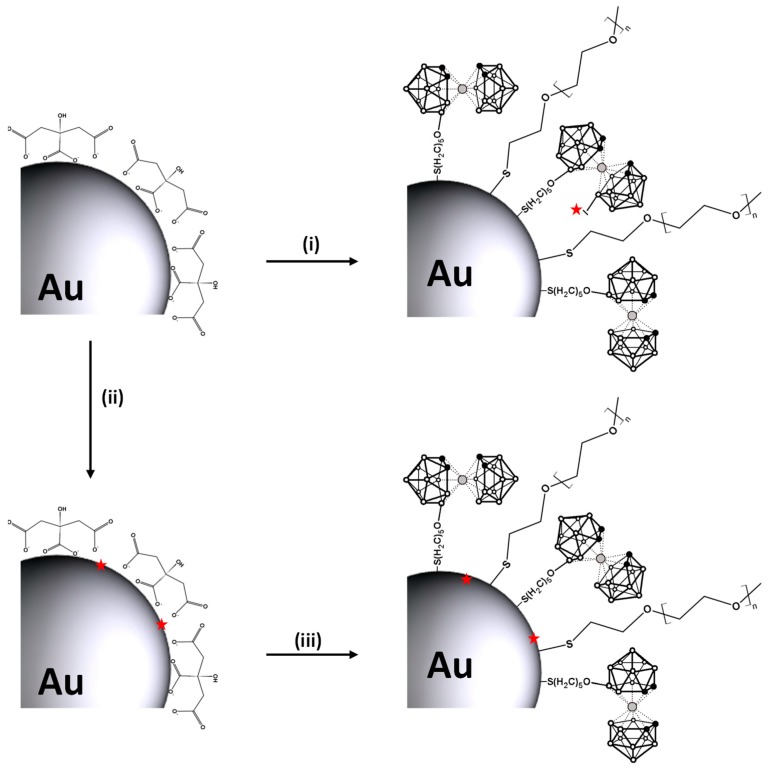
Schematic representation of the different routes followed for the preparation of radiolabelled PEG-AuNPs@[**4**]^−^. (i) PEG-SH (5 Kda), ^124^I-[**6**]^−^ + [**4**]^−^; (ii) [^124^I]NaI; (iii) PEG-SH (5 Kda), [**4**]^−^. The red star stands for the radiolabel (^124^I).

Here, the decided to tackle the radiolabelling of PEG-AuNPs@[**4**]^−^ by using two previously reported approaches, in order to incorporate the positron emitter ^124^I (half-life of 4.2 days) either at the shell or at the core of the NPs (Figure 3). 

In the first approach ^124^I-[**6**]^−^ was prepared by isotopic exchange, following a methodology previously described in our group [54]. Incorporation ratios of the radiolabel of 69 ± 6% were achieved when the reaction was conducted at 100 °C for 5 min, as determined by HPLC using radiometric detection. These values are in good agreement with those previously described [54]. Purification by semi-preparative HPLC resulted in a solution of chemically and radiochemically pure compound ^124^I-[**5**]^−^ with radiochemical yield (non-decay corrected) of ca. 60%. Hydrolysis to achieve ^124^I-[**6**]^−^ was finally carried out before approaching the preparation of radiolabelled NPs and the resulting compound was used without further purification. Subsequent preparation of functionalized, radiolabelled AuNPs was carried out following the same process described above, but compound [**4**]^−^ was spiked with ^124^I-[**6**]^−^. Radiolabelling efficiency, calculated as the amount of radioactivity present in the NPs related to the starting amount of ^124^I-[**6**]^−^ was 55%, suggesting that compounds [**4**]^−^ and ^124^I-[**6**]^−^ are similarly attached to the surface of AuNPs. Analysis of the resulting NPs after complete decay using DLS and TEM showed equivalent properties to those obtained for the non-labelled NPs (results not shown). 

The second approach for the preparation of radiolabelled AuNPs was based on the absorption of radioiodine on the surface of the gold core [55,56]. Low incorporation efficiencies (<10%) were achieved when functionalized NPs were incubated with the radionuclide. These results are not in agreement with previously reported works, in which almost quantitative labelling yields were obtained [55,56]. However, incubation of citrate-stabilized NPs with radioiodine resulted in incorporation ratios >95% in short times (10 min), results that are more in line with those previously reported in the literature. Hence, the presence of the COSAN anions on the surface of the NPs seems to hamper the absorption of radioiodide on the surface, probably due to electrostatic repulsion. In view of these results, we decided to incorporate the radiolabel on citrate-stabilized NPs and incorporate both the PEG and COSAN derivatives in a second step (Figure 3). 

**Figure 4:**
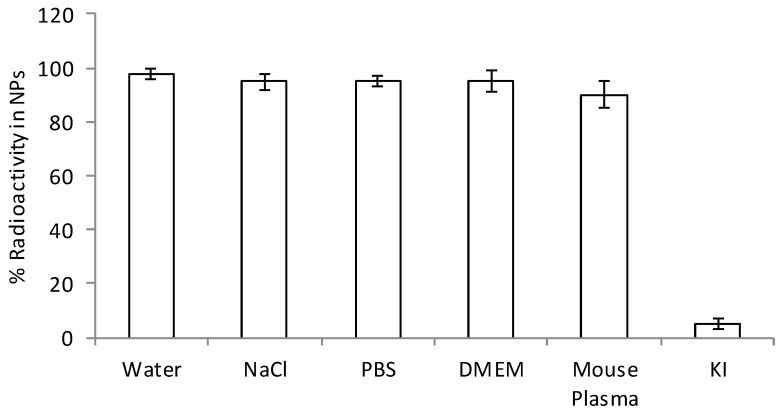
Radiochemical stability of core-labelled PEG-AuNPs@[**4**]^−^ in different media; NaCl: physiologic saline solution (0.9%); PBS: 10 mM phosphate buffered solution; DMEM: Culture media, cocktail of 8 salts, 15 amino acids, 8 vitamins and glucose; KI: potassium iodide solution (10 µM).

One of the main limitations of this second labelling approach is the risk of detachment of the absorbed radionuclide. In order to evaluate the radiochemical stability of core-labelled [^124^I]PEG-AuNPs@[**4**]^−^, these were incubated in different media, including water, saline, PBS (10 mM), DMEM (cell culture media), mouse plasma and potassium iodide (10 µM). After incubation at 37 °C for 24 h, the NPs were separated from the media by centrifugal filtration and washed three times, and the radiochemical stability was determined as the ratio between the amount of radioactivity in the NPs and the total amount of radioactivity (NPs + media + washings). Good radiochemical stability (>90%) was observed in all media except potassium iodide (Figure 4), thus confirming the chemisorption of ^124^I and the suitability of the labeling strategy for in vivo studies. 

Noteworthy, the structural (size and aspect) and chemical properties (composition in terms of boron load) remained unaltered after incorporation of the radiolabel either at the core or at the shell, confirming the suitability of both labelling methods to tackle in vivo experiments. 

### 2.4. In Vivo Imaging Studies

The capacity of the newly developed AuNPs ([^124^I]PEG-AuNPs@[**4**]^−^) to accumulate in tumour tissue was investigated in a subcutaneous mouse model generated by inoculation of HT1080 cells in nude mice. Traditionally, BNCT has been investigated mainly for brain (gliomas) and head & neck cancers [57]. However, this therapeutic approach can in principle be evaluated in any cancer type, and those cancers with high mortality or lack of efficient therapeutic alternatives are worth to be assayed. Hence, we decided to investigate human fibrosarcoma, which is rare but highly aggressive, and unlike other cancers, metastasizes at early stages. The standard therapy, which includes surgical resection and adjuvant chemotherapy, is ineffective due to local recurrence and distant metastasis [58].

Imaging studies performed in mice enabled the determination of the biodistribution pattern of the labelled particles. Additionally, the inclusion of the label in the two different positions (the core and the shell) was used to gain information about the stability of the core-shell structure, an issue that is rarely tackled in preclinical in vivo investigations. With that aim, animals were injected with the labelled particles and images were acquired immediately after administration (total acquisition time of 60 min). Additionally, 30 min static acquisitions were recorded also at t = 10, 24, 72 and 144 h post-administration (Figure 4). 

**Figure 5:**
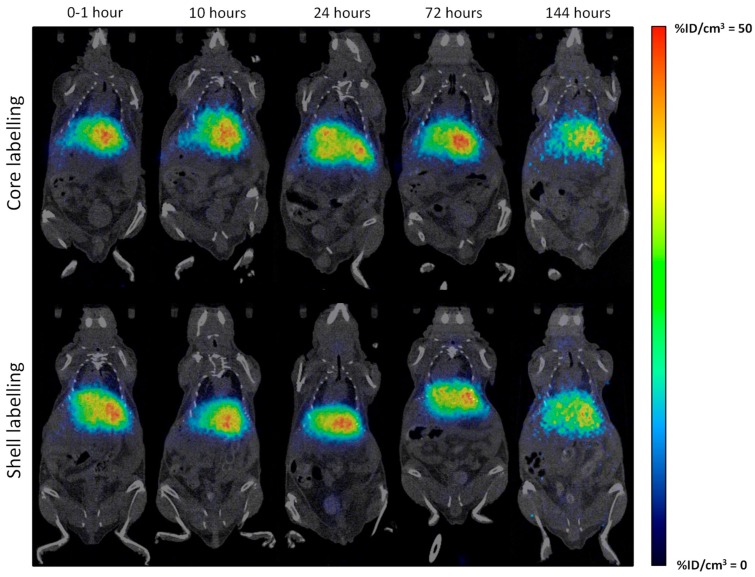
PET images (coronal projections) obtained at different time points after intravenous administration of [^124^I]PEG-AuNPs@[**4**]^−^ labelled at the core (top) and at the shell (bottom). PET images have been coregistered with representative CT slices for localization of the radioactive signal. The image labelled as “0–1 h” corresponds to the image obtained immediately after administration of the labelled NPs (imaging time = 1 h). Values expressed as %ID/cm^3^.

**Figure 6:**
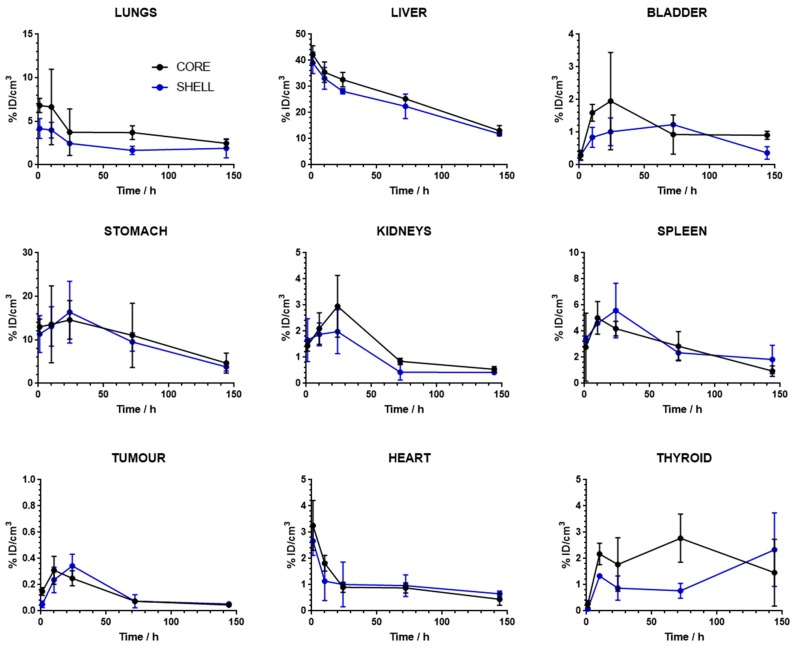
Concentration of radioactivity in the different organs as determined from PET images using the two radiolabelling approaches. Values are expressed as %ID/cm^3^. Values correspond to mean ± standard deviation, n = 2 per labelling strategy and time point.

Visual inspection of the images (Figure 5) showed a similar biodistribution pattern for both PEG-AuNPs@[**4**]^−^, irrespectively of the position of the label, with high accumulation in the liver already at short times after administration (0–1 h). Interestingly, no accumulation was detected in the thyroid gland, confirming the lack of de-iodination during in vivo experimentation. Delineation of volumes of interest (VOIs) in major organs (lungs, liver, bladder, stomach, kidneys, spleen, tumour, heart and thyroid gland) and determination of the uptake as % of injected dose per cm^3^ tissue (%ID/cm^3^, see Figure 6) revealed similar profiles for both NPs, this confirming the in vivo stability of the core-shell structure. Major accumulation at short times after administration was observed in the liver. For this organ, values at the first time point were 42.2 ± 4.8 and 39.1 ± 5.8 %ID/cm^3^ for core and shell labelling, respectively. These values progressively decrease with time to reach values close to 10 %ID/cm^3^ at t = 144 h, confirming the progressive elimination of the NPs. Accumulation in the stomach peaked at t = 24 h after administration, with progressive elimination from this organ at later time points. Accumulation in the lungs was also significant. Of note, the detection of radioactivity in the heart reflects the presence of labelled NPs in the bloodstream, even at long times after administration. The presence of radioactivity in the bladder at long times after administration confirms slow elimination via urine. This result, together with the detection of radioactive signal in the thyroid gland, suggests the progressive (although very slow) detachment of the radiolabel in both cases (core and shell labelling). Accumulation in the thyroid gland seems to occur faster for core-labelled particles, although these values are subjected to a significant error due to difficulties in delineation of the VOIs, and hence these values should be only considered qualitatively. 

**Figure 7:**
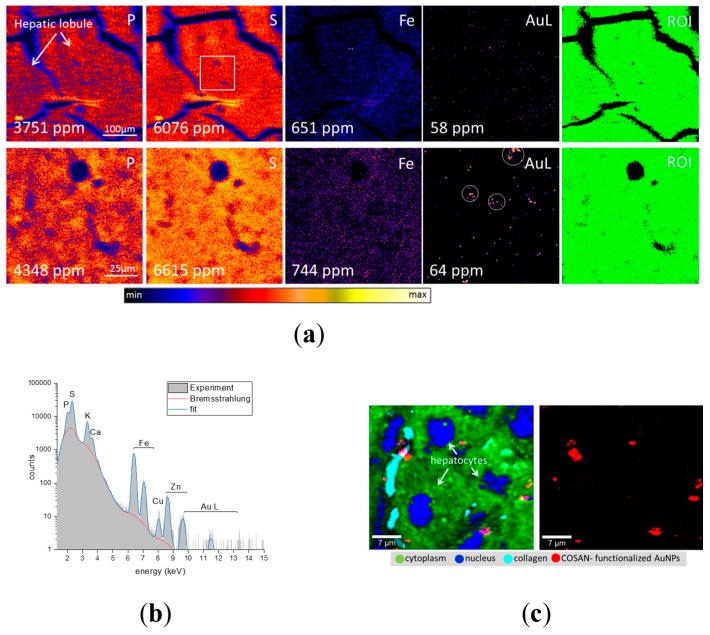
(**a**) µPIXE images of intrinsic tissue elements (P, S, Fe) as well as Au in mouse liver at t = 72 h after intravenous administration of [^124^I]PEG-AuNPs@[**4**]^−^. The area in the white rectangle was subsequently scanned with higher resolution. The concentration of elements was calculated from ROIs shown in green on the right side. White arrows denote hepatic lobules. White circles show small gold clusters; (**b**) PIXE spectrum extracted from ROIs; (**c**) CRM images of hepatocytes and distribution of [^124^I]PEG-AuNPs@[**4**]^−^ in in mouse liver at t = 72 h after intravenous administration of [^124^I]PEG-AuNPs@[**4**]^−^. The color-coded image (left) shows the overlapping of nucleus, cytoplasm, collagen and COSAN- functionalized Au NPs.

The accumulation of NPs in the liver observed in vivo was confirmed by ex vivo analysis. With that aim, animals were sacrificed after the last imaging session and the liver was investigated using ion beam microscopy (IBM) and confocal Raman microspectroscopy (CRM) (Figure 7). These label-free techniques have proven efficient for simultaneous quantification and visualization of different NPs in biological environments [59,60,61,62,63,64,65,66], both in vitro and in vivo. The µPIXE spectrum extracted from the region of interest (ROI) in Figure 7b reveals X-ray emission of intrinsic tissues elements, i.e., P, S, Ca etc., as well as of gold. Characteristic Lα, Lβ and Lγ X-ray lines of gold at 9.7 keV, 11.4 keV and 13.4 keV were detected in the ROIs shown as green regions in Figure 7a. The structure of several connected hepatic lobules could be identified due to P and S distribution in the PIXE images. PIXE maps of Au distribution showed a rather homogenous distribution all over those lobules. However, a high resolution image with a size of 100 × 100 µm revealed small gold clusters in the hepatocytes (white circles in Figure 7a). 

CRM imaging was additionally used to visualize the distribution pattern of NPs in the liver at subcellular level (Figure 7c). Au NPs were localized in hepatic lobules by using photoluminescence signals of Au. Cytoplasm, nuclei and collagen were visualized on the basis of Raman fingerprint regions of biomolecules [67,68,69], which are predominantly present in the corresponding constituents of cell. As it can be seen, lobules are surrounded by supporting connective tissue, which was identified in CRM imaging by the presence of collagen. Au NPs were found in the cytoplasm of hepatocytes as well as in close vicinity to their nuclei. Some Au NP aggregates were also detected between adjacent lobules showing a co-localization with the connective tissue. 

In general terms, our biodistribution data correlates well with previous results obtained with negatively charged NPs. In general, it has been found that, upon single administration, spherical AuNPs localize in liver, kidneys, spleen and lungs, with this phenomenon being size- and shape-dependent. For NPs in our size range, major accumulation should be expected in liver, spleen and lungs [70]. 

Unfortunately, and in spite of the long circulation time of a fraction of the NPs, our NPs did not show significant accumulation in the tumour, with maximum values below 0.5 %ID/cm^3^ irrespective of the labelling approach achieved at 12 h (core labelling) and 24 h (shell labelling) after administration. Such accumulation progressively decreased with time and is almost undetectable at t > 72 h. The decrease in the amount of radioactivity observed in the tumour could be a consequence of the AuNPs extravasating the tumour again due to high tumour pressure, although internalisation of the NPs in the tumour cells followed by degradation and free iodine release seems a more plausible hypothesis, and explains the accumulation of radioactivity observed in thyroid gland and urine. 

Considering that the boron load of our NPs is ca. 9% and that the administered dose of NPs was 150 µg (ca. 7.5 mg NP/kg body weight; 0.675 mg B/kg body weight), the boron accumulation in the tumour is close to 0.06 µg B/g of tumour. This value is by far too low to plan any therapeutic experiment and it is inferior to those reported in the literature using other nanocarriers.

The most similar boron carriers described in the literature have been recently reported by Wu et al. [36]. In this work, the authors prepared targeted (functionalised with an anti-HER2 antibody; particle size 54.48 ± 14.72 nm, as determined by DLS) and non-targeted (57.59 ± 13.90 nm, as determined by DLS) carborane-substituted AuNPs using commercially available citrated-capped AuNPs which were subjected to PEGylation, azide addition, and boron-cage modification on the surface. The resulting AuNPs were radiolabelled with ^123^I, a gamma emitter with a half-life of 13.22 h which enables in vivo imaging using single photon emission computerised tomography (SPECT). As in our results, high accumulation was found in the liver. However, both targeted and non-targeted AuNPs reported in this previous work showed significant tumor accumulation in a mouse model of gastric cancer over-expressing HER-2. According to in vivo data, accumulation in the tumor was 48.32 ± 3.11 %ID/mL and 7.43 ± 0.28 %ID/mL for the targeted and non-targeted AuNPs, respectively, at 12 h post injection, with tumor-to-muscle (T/M) ratios of 12.02 ± 0.94 and 1.91 ± 0.17, respectively. The first value progressively decreased with time, suggesting the internalisation of the targeted particles, which could be transported to lysosomes and degraded with the consequent release of free iodine, which was finally accumulated in the thyroid gland. On the contrary, the tumor uptake values remain almost unaltered for the non-targeted particles in the 12–36 h time window, while T/M values significantly increase at t = 36 h. These previous results are more promising than our data, including those obtained with non-targeted particles, in which the tumor accumulation is ca. 15 times higher than the accumulation observed with our particles. The reason behind these differences is at the moment unclear, although it might be related to differences in particle size, surface charge (values not reported in [36]) or animal model and tumor size. The latter may have a huge impact in the results, as different pathophysiological features which vary among tumor models regulate the efficiency of EPR-mediated tumour targeting. These include intratumoural blood flow, angiogenic vascular permeability, tumour microenvironment, interstitial pressure and lymphatic drainage [71]. 

Other nanocarriers assayed so far in the context of BNCT are liposomes, which deserve special mention as they have been widely used in cancer therapy. For example, a delivery system consisting of polyethylene-glycol (PEG) binding liposomes (DPPC/cholesterol/DSPC-PEG2000) loaded with ^10^B-enriched BSH showed tumour growth suppression in a mouse xenograft model of human pancreatic carcinoma [72]. The amount of ^10^B in the tumour was measured by prompt gamma-spectroscopy and reached values of 2.37 µg/g of tumour at 24 h after administration. In more recent works, BSH-encapsulating liposomes containing lipophilic boron compounds embedded in the bilayer have been developed and evaluated as BNCT agents in a mouse xenograft model of colon cancer. The amount of boron accumulated in the tumour was evaluated by inductively coupled plasma atomic emission spectroscopy (ICP-AES) at different administered doses. Maximum tumour accumulation was achieved at 36 h after administration, with values close to 175 µg/g of tumour when the administered dose was 50 mg B/kg, corresponding to 17.5 %ID/g (assuming a mouse weight of 20 g) [73]. The tumour accumulation was exceedingly higher that that achieved in our case (ca. 3000 times higher) although the administered dose was also ca. 250 times higher).

Other nanocarriers such as carbon nanotubes (CNTs) have been also investigated. In one of the pioneering works, substituted carborane cages were attached to the side walls of single wall carbon nanotubes (SWCNTs) and evaluated in a xenograft mouse model of mammary carcinoma, resulting in tumour accumulation values close to 23 µg/g of tumour as determined by inductively coupled plasma-optical emission spectrometry (ICP-OES) [74].

Inorganic NPs have been also assayed as BNCT drug candidates. For example, magnetic NPs functionalised with *o*-carborane were prepared and evaluated in a mouse model of breast cancer. Taking advantage of the magnetic properties of the particles, high tumour accumulation (51.4 μg/g tumor) could be obtained under the influence of an external magnetic field (1.14 T), corresponding to ca. 3 %ID/cm^3^ [75]. However, the application of this approach to real clinical scenarios might be unfeasible, especially in the presence of metastasis.

Other examples of nanosystems engineered for boron delivery into tumour tissue have been reported and recently reviewed [76]. Different degrees of tumour accumulation have been achieved in different tumour models and experimental approaches. However, the diversity of tumour models and experimental conditions limits direct comparison with our results, as tumour accumulation, which ultimately relies solely in EPR effect, is severely affected by the physiology of the tumour.

To the best of our knowledge, our work constitutes the first example of in vivo evaluation of gold nanoparticles functionalised with COSAN moieties as boron carriers for BNCT. In spite of the fact that poor tumour accumulation was achieved, appropriate redesign of the core in terms of size and shape should lead to improved results. We anticipate that the use of smaller particles or nanorod-shaped particles should lead to lower sequestration by the liver as previously reported [70], thus increasing circulation time and facilitating tumour accumulation. Additionally, gold nanorods present the well-known ability to absorb near infrared (NIR) light, enabling photo-thermal therapy and paving thus the way towards combined therapies. Last but not least, our labelling strategies enable the determination of the location of the NPs in vivo, in real time and with high sensitivity, reducing thus the number of animals required for the fast investigation of new nanosystems as BNCT drug candidates. 

## 3. Materials and Methods 

### 3.1. Reagents

Cesium cobalt(III) *bis*(dicarbollide) (COSAN) (Katchem Ltd., Prague, Czech Republic), Gold(III) chloride trihydrate (HAuCl_4_·3H_2_O, Aldrich, Madrid, Spain), Sodium citrate (Sigma-Aldrich, St. Louis, MO, USA) and poly (ethylene glycol) methyl ether thiol (MW 5000, Sigma-Aldrich) were used as purchased. All other reagents and anhydrous solvents, stored over 4 Å molecular sieves, were purchased from Aldrich Chemical Co. (Madrid, Spain) and used without further purification. HPLC grade ethanol, methanol, and acetonitrile were purchased from Scharlab (Sentmenat, Barcelona, Spain).

Experiments were carried out, except when noted, under a dry, oxygen-free dinitrogen atmosphere. Column chromatography was performed using silica gel 60 (Scharlab). Analytical thin layer chromatography (TLC) measurements were conducted with silica gel 60 F_254_ plates (Macherey-Nagel, Hoerdt, France); and the spots were visualized under UV lamp. Compound **2** was prepared and characterized according to the previously reported protocol [40].

### 3.2. Instrumentation

The ^1^H-NMR (500 MHz), ^13^C-NMR (126 MHz) and ^11^B-NMR (160 MHz) spectra were recorded on a 500-MHz Avance III spectrometer (Bruker BioSpin, Switzerland). All NMR spectra were performed in deuterated solvents at 22 °C. The ^11^B-NMR shifts were referenced to external BF_3_·OEt_2_, while the ^1^H and ^13^C-NMR shifts were referenced to SiMe_4_. Chemical shifts are reported in units of parts per million downfield from the reference, and all coupling constants are reported in Hertz. 

UPLC/ESI-MS analyses were performed using an AQUITY UPLC separation module coupled to a LCT TOF Premier XE mass spectrometer (Waters, Manchester, UK). An Acquity BEH C18 column (1.7 µm, 5 mm, 2.1 mm) was used as stationary phase. The elution buffers were A (water and 0.1% formic acid) and B (Methanol and 0.1% formic acid). The column was eluted with a gradient: t = 0 min, 95% A, 5% B; t = 0.5 min, 95% A, 5% B; t = 5.5 min, 25% A, 75% B; t = 16 min, 1% A, 99% B; t = 20 min, 1% A, 99% B. Total run was 20 min, injection volume was 5 µL and the flow rate 300 µL/min. The detection was carried out in both negative and positive ion mode, monitoring the most abundant isotope peaks from the mass spectra (M − H^+^) or (M + H^+^).

Transmission electron microscopy (TEM) was performed using a JEOL JEM-1400 plus microscope (Jeol, Tokyo, Japan) working at 120 kV. The carbon film of copper grids (CF400-Cu) was treated under air plasma in a glow discharge system (K100X, Emitech, Kent, UK, 40 mA during 2 min) just before sample preparation. For TEM examinations, a single drop (1 μL) of the NPs solution was placed onto a copper grid coated with a carbon film (Electron Microscopy Sciences, Hatfieled, PA, USA). After 1 min, the drop was removed with filter paper and the sample was incubated with 3 μL of uranyl acetate 0.5% (3 min).

XPS experiments were performed in a SPECS Sage HR 100 spectrometer (Berlin, Germany) with a non-monochromatic X ray source (aluminium Kα line of 1486.6 eV energy and 252 W), placed perpendicular to the analyser axis and calibrated using the 3d5/2 line of Ag with a full width at half maximum (FWHM) of 1.1 eV. The selected resolution for the spectra was 15 eV of Pass Energy and 0.15 eV/step. All measurements were made in an ultra-high vacuum (UHV) chamber at a pressure around 6 × 10^−8^ mbar. An electron flood gun was used for charge neutralisation. Gaussian Lorentzian functions were used for fittings (after a Shirley background correction) where the FWHM of all the peaks were constrained while the peak positions and areas were set free. Main C1s peak was used for charge reference and set at 284.8 eV.

ICP-MS measurements were performed on a Thermo iCAP Q ICP-MS (Thermo Fisher Scientific GmbH, Bremen, Germany). An ASX-560 autosampler was coupled to the ICP-MS (CETAC Tech, Omaha, NE, USA). UV-Vis spectra were measured in an 8453 UV-Vis diode-array spectrophotometer (Agilent, Santa Clara, CA, USA). DLS and ξ-potential measurements were performed using a Malvern Zetasizer Nano ZS system (Malvern Instruments, Malvern, UK). The particle size measurement settings were: 3 measurements/14 runs/10s in scattered mode at 173° angle. Measurements were conducted at T = 25 °C and neutral pH. Raman characterization was performed with an InVia Raman Microscope (Renishaw, Gloucestershire, UK), using a 633 nm laser (50% power) with 10× objective. 

### 3.3. Chemistry

#### 3.3.1. Synthesis of [**3**]^−^

To a solution of **2** (750 mg, 1.209 mmol) in DMF (8 mL), potassium thioacetate (165.7 mg, 1.451 mmol) was added and stirred at room temperature for 14 h. For the workup, 50 mL of water was added and extracted with ethyl acetate (3 × 50 mL); the organic layers were combined and washed with cold water and brine solution before drying over anhydrous sodium sulphate. The solvent was evaporated and the crude was purified using column chromatography (silica gel, 10% MeOH in dichloromethane) to yield a [**3**]^−^ as yellow solid (705 mg, 84%). ^1^H-NMR (methanol-*d*_4_) δ 4.15 [C_c_–*H*, 2H, s], 4.07 [C_c_–*H*, 2H, s], 3.49 [C*H*_2_O, 2 H, t], 2.87 [C*H*_2_-S, 2 H, t], 2.31 [C*H*_3_, 3 H, s], 1.59–1.54 [C*H*_2_-CH_2_-O, 2 H, m], 1.52–1.47 [C*H*_2_-CH_2_S, 2 H, m], 1.42–1.37 [C*H*_2_-CH_2_CH_2_O, 2 H, m]; ^11^B-NMR (methanol-*d*_4_) δ 23.03[1B, s], 4.63 [1B, d, ^1^J(B-H) = 140.3], 0.48 [1B, d, ^1^J(B-H) = 143.3], −2.10 [1B, d, ^1^J(B-H) = 142.8], −4.59[2B, d, ^1^J(B-H) = 138.7], −8.46 [6B, td, *J* 162.8, 150.9, 63.5], −17.22 [2B, d, ^1^J(B-H) = 154.4], −20.42 [2B, d], -22.59 [1B, d], −28.26 [1B, d, ^1^J(B-H) = 159.4]; ^13^C-NMR (methanol-*d*_4_): 188.24, 68.93, 53.54, 46.70, 30.89, 29.21, 29.14, 28.61, 25.10; LCMS (ESI) Experimental [M]^−^ *m/z* = 482.66 (theoretical [M]^−^ *m/z* = 482.98).

#### 3.3.2. Synthesis of [**4**]^−^

To a solution of [**3**]^−^ (890 mg, 1.440 mmol) in methanol (30 mL), sodium methoxide (77.8 mg, 1.440 mmol) was added and stirred at room temperature for 14 h. For the workup, reaction mixture was neutralized with IR-120 resin (3 g), filtered and washed with 10 mL of methanol. Combined methanol layers were concentrated and purified using column chromatography (silica gel, 12% MeOH in dichloromethane) to yield [**4**]^−^ as a yellow solid (505 mg, 60.5%). ^1^H-NMR (methanol-*d*_4_) δ 4.14 [C_c_–*H*, 2H, bs], 4.06 [C_c_–*H*, 2H, bs], 3.51 [C*H*_2_O, 2 H, t], 2.50 [C*H*_2_-S, 2 H, t], 1.60 [C*H*_2_-CH_2_-O, 2 H, p], 1.58, 1.53 [C*H*_2_-CH_2_S, 2 H, dq], 1.42 [C*H*_2_-CH_2_CH_2_O, 2 H, qd]; ^11^B-NMR (methanol-*d*_4_) δ 23.02 [1B, s], 4.64 [1B, d, ^1^J(B-H) = 140.0], 0.52 [1B, d, ^1^J(B-H) = 142.0], −2.11 [1B, d, ^1^J(B-H) = 144.5], −4.56 [2B, d, ^1^J(B-H) = 142.4], −7.41 [6B, m], −17.20 [2B, d, ^1^J(B-H) = 157.7], −20.46 [2B, d, ^1^J(B-H) = 159.0], −22.05 [1B, d], −28.22 [1B, d, ^1^J(B-H) = 174.3]; ^13^C-NMR (methanol-*d*_4_) δ 69.08, 53.46, 46.70, 33.90, 30.83, 24.71, 23.65. LCMS (ESI) Experimental [M]^−^ *m/z* = 440.52 (theoretical [M]^−^ *m/z* = 440.94).

#### 3.3.3. Synthesis of [**5**]^−^

To a solution of [**3**]^−^ (50 mg, 0.081 mmol) in acetonitrile (2 mL), chloramine-T (37 mg, 0.129 mmol), sodium iodide (15 mg, 0.097 mmol) and acetic acid (130 μL) were added. The reaction mixture was allowed to stir at room temperature for 10 h. For the workup, 20 mL of water were added and extracted with ethyl acetate (3 × 15 mL), the organic layers were combined and washed with brine solution before drying over anhydrous sodium sulphate. The solvent was evaporated and the crude was purified using column chromatography (silica gel, 10% MeOH in dichloromethane) to yield [**5**]^−^ as a yellow solid (25 mg, 41.6%). ^1^H-NMR (methanol-*d*_4_) δ 4.27 [C_c_–*H*, 2H, s], 4.13 [C_c_–*H*, 2H, s], 3.37 [C*H*_2_O, 2 H, t], 2.85 [C*H*_2_-S, 2 H, t], 2.31 [C*H*_3_, 3 H, s], 1.55 [C*H*_2_-CH_2_-O, 2 H, m], 1.45 [C*H*_2_-CH_2_S, 2 H, m], 1.36 [C*H*_2_-CH_2_CH_2_O, 2 H, m]; ^11^B-NMR (methanol-*d*_4_) δ 21.47 [1B, s], −0.42[2B, d, ^1^J(B-H) = 145.4], −4.39 [1B, s], −5.71 [4B, d, ^1^J(B-H) = 140.0], −7.27 [4B, d, ^1^J(B-H) = 152.8], −18.07 [2B, d, ^1^J(B-H) = 152.4], −20.04 [2B, d, ^1^J(B-H) = 161.3], −23.45 [1B, d, ^1^J(B-H) = 161.3], −27.37 [1B, d, ^1^J(B-H) = 170.4]; ^13^C-NMR (CDCl_3_) δ 191.05, 70.24, 58.99, 56.52, 34.78, 33.10, 32.97, 32.52, 29.24. LCMS (ESI) Experimental [M]^−^ *m/z* = 606.6 (theoretical [M]^−^ *m/z* = 607.6).

#### 3.3.4. Synthesis of [**6**]^−^

To a solution of [**5**]^−^ (9 mg, 0.0144 mmol) in methanol (3 mL), sodium methoxide (1 mg, 0.0144 mmol) was added and the resulting solution was allowed to stir at room temperature for 14 h. For the workup, the reaction mixture was neutralized with IR-120 resin (100 mg), filtered and washed with 2 mL of methanol. The combined methanol layer was concentrated and the residue was purified using column chromatography (silica gel, 12% MeOH in dichloromethane) to yield [**6**]^−^ as a yellow solid (4.5 mg, 55%). ^1^H-NMR (methanol-*d*_4_) δ 4.28 [C_c_–*H*, 2H, s], 4.14 [C_c_–*H*, 2H, s], 3.40 [C*H*_2_O, 2 H, t], 2.67 [C*H*_2_-S, 2 H, t], 1.66 [C*H*_2_-CH_2_-O, 2 H, m], 1.46 [C*H*_2_-CH_2_S, 2 H, q], 1.38 [C*H*_2_-CH_2_CH_2_O, 2 H, m]; ^11^B-NMR (mMethanol-*d*_4_) δ 21.55 [1B, s], −0.34 [2B, d, ^1^J(B-H) = 146.7], −4.32 [1B, s], −5.57 [4B, d, ^1^J(B-H) = 170.0], −6.90 [4B, d], −17.94 [2B, d, ^1^J(B-H) = 167.3], −20.58 [2B, d, ^1^J(B-H) = 153.2], −23.13 [2B, d, ^1^J(B-H) = 266.4], −26.79 [2B, d]; ^13^C-NMR (methanol-*d*_4_) δ 68.68, 56.57, 53.91, 38.38, 31.04, 28.70, 24.81. LCMS (ESI) Experimental [M]^−^ *m/z* = 566.64 (theoretical [M]^−^ *m/z* = 566.84).

### 3.4. Radiochemistry

#### 3.4.1. Synthesis of ^124^I-[**5**]^−^

Acetonitrile (200 μL) was added to Na [^124^I]I (50 µL, 37 MBq) and the resulting solution was introduced in a 2.5 mL conic vial. The solvent was evaporated to dryness (100 °C, 5 min, constant helium flow at 20 mL/min) and 1 mg of [**5**]^−^ dissolved in acetonitrile (100 μL) was added together with *trans*-*bis*(acetate)*bis*[*o*-(di-*o*-tolylphosphino)benzyl] dipalladium (II) (Herrmann’s catalyst, HC, 0.1 mg, 0.101 μmol) dissolved in toluene (100 µL). The reaction mixture was heated at 100 °C for 5 min, the solvent was removed under a constant helium flow and the resulting solid was dissolved in 0.5 mL acetonitrile and 30 mL MQ water. The crude solution was passed through a C-18 cartridge (Sep-Pak^®^ Light, Waters, Milford, MA, USA) and washed with MQ water (5 mL × 2) to remove free iodine-124. The desired labelled compound (^124^I-[**5**]^−^) was eluted from the cartridge with ethanol (500 μL) and the solvent was evaporated to dryness. Quality control was performed by radio-HPLC after diluting the solid residue with mobile phase. Analytical conditions were: Stationary phase: Mediterranea Sea18 column (4.6 × 150 mm, 5 μm particle size, Teknokroma, Barcelona, Spain); mobile phase A: 0.1 M ammonium formate (AMF) buffer pH = 3.9; B: acetonitrile; flow rate = 1 mL/min; gradient: 0 min: 60% A- 40% B; 2 min: 60% A- 40% B; 6 min: 20% A- 80% B; 14 min: 0% A- 100% B; 16 min: 0% A- 100% B; 18 min: 60% A- 40% B; 20 min: 60% A- 40% B (retention time: 11.5 min). 

#### 3.4.2. Synthesis of ^124^I-[**6**]^−^

Dry ^124^I-[**5**]^−^ obtained from the previous step was dissolved in 250 μL of methanol. Sodium methoxide (2 mg) was added and stirred at room temperature for 6 h. After Complete hydrolysis, confirmed by analytical radio-HPLC, reformulation was carried out by dilution with water, retention on a C-18 cartridge (Sep-Pak^®^ Light, Waters), further elution with ethanol (500 μL, Sigma-Aldrich) and evaporation to dryness. Quality control was performed by HPLC. Analytical conditions were: Stationary phase: Mediterranea Sea18 column (4.6 × 150 mm, 5 μm particle size, Teknokroma); mobile phase A: 0.1 M ammonium formate (AMF) buffer pH = 3.9; B: acetonitrile; flow rate = 1 mL/min; gradient: 0 min: 60% A- 40% B; 2 min: 60% A- 40% B; 6 min: 20% A- 80% B; 14 min: 0% A- 100% B; 16 min: 0% A- 100% B; 18 min: 60% A- 40% B; 20 min: 60% A- 40% B (retention time: 13.1 min).

### 3.5. Preparation of AuNPs

#### 3.5.1. Synthesis of Citrate-Stabilized Gold NPs (CIT-AuNPs)

AuNPs-Citrate with an averaged diameter 18–20 nm and with Au concentration of 1 mg/mL were synthesized following the Turkevich method [44]. In brief, 97.1 mg (0.33 mmol) trisodium citrate dihydrate were dissolved in 150 mL water (concentration = 2.2 mM) and the solution was heated to reflux in a 250 mL three-necked flask equipped with a Dimroth condenser. The boiling time before addition of the precursor was 15 min. 1 mL precursor solution (HAuCl_4_∙3H_2_O in water, 25 mM) was then quickly injected under rapid stirring. When the colour of the solution changed to the characteristic wine-red, which indicates formation of AuNPs, the heating-mantle was switched off but not removed until the temperature of the solution was 70 °C. The resulting NPs were centrifuged at 12,000× *g* for 20 min to remove free citrate and resuspended in ultrapure water.

#### 3.5.2. Synthesis of PEG-Stabilized, COSAN-Functionalized AuNPs (PEG-AuNPs@[**4**]^−^)

AuNPs-Citrate prepared as described above (2 mL) were place into a vial. Then 100 μL of PEG (3 mg/mL) were slowly added under vigorous stirring. After 15 min, 100 μL of a fresh solution of [**4**]^−^ in ethanol (3 mg/mL) were quickly added and stirring was maintained for 2 h. The resulting NPs were centrifuged at 12,000× *g* for 25 min and resuspended in ultrapure water three times.

#### 3.5.3. Synthesis of PEG-AuNPs@[**4**]^−^ Labelled at the Core 

AuNPs-Citrate prepared as described above (2 mL) were placed into a vial. [^124^I]NaI (15 μL, solution in 0.1 M NaOH) was added and the solution was stirred for 10 min. Then, PEG (100 μL, 3 mg/mL) was slowly added under vigorous stirring. After 15 min, 100 μL of a fresh solution of [**4**]^−^ in ethanol (3 mg/mL) were quickly added and stirring was maintained for 2 h. The resulting NPs were centrifuged at 12,000× *g* for 25 min and resuspended in ultrapure water three times. Radiochemical incorporation ratios around 98% were obtained.

#### 3.5.4. Synthesis of PEG-AuNPs@[**4**]^−^ Labelled at the Shell 

2 mL of AuNPs-Citrate prepared as described above were placed into a vial. Then, PEG (100 μL, 3 mg/mL) was slowly added under vigorous stirring. After 15 min, 100 μL of a fresh solution of [**4**]^−^ in ethanol (3 mg/mL) and freshly prepared ^124^I-[**6**]^−^ (22.2 MBq) dissolved in ethanol (15μL) were quickly added and stirring was maintained for 2 h. The resulting NPs were centrifuged at 12,000× *g* for 25 min and resuspended in ultrapure water three times. Radiochemical incorporation ratios around 55% were obtained.

### 3.6. In Vivo Studies

Animals were maintained and handled in accordance with the Guidelines for Accommodation and Care of Animals (European Convention for the Protection of Vertebrate Animals Used for Experimental and Other Scientific Purposes). All animal procedures were performed in accordance with the Spanish policy for animal protection (RD53/2013), which meets the requirements of the European Union Animal Directive (2010/63/EU). Experimental procedures were approved by Ethical Committee of CIC biomaGUNE.

PET studies with labelled NPs were carried out in mice (n = 2 per compound) using an eXplore Vista-CT small animal PET-CT system (GE Healthcare, Chicago, IL, USA). Anaesthesia was induced with 3% isoflurane and maintained by 1.5 to 2% of isoflurane in 100% O_2_. For intravenous administration of the radiotracer, the tail vein was catheterized with a 24-gauge catheter and the labelled NPs (3.8 ± 0.6 MBq for NPs labelled at the core; 2.7 ± 0.3 MBq for NPs labelled at the shell, volume = 150 μL) were injected concomitantly with the start of a PET dynamic acquisition. Mice were kept normothermic throughout the scans using a heating blanket (Homeothermic Blanket Control Unit, Bruker BioSpin GmbH, Karlsruhe, Germany). 

Whole body scans were acquired just after administration during 60 min. At time points 10 h, 24 h, 72 h and 144 h, 30 min acquisitions were performed. All the scans were recorded in the 400–700 KeV energetic window. CT acquisitions were also performed at the end of each PET scan, providing anatomical information for unambiguous localisation of the radioactive signal. After the last imaging session, animals were sacrificed and the liver was harvested for further processing.

PET images were reconstructed (decay and CT-based attenuation corrected) with filtered back projection (FBP) using a Ramp filter with a cut off frequency of 1 Hz. Images were analysed using PMOD image analysis software (version 3.4, PMOD Technologies Ltd., Zürich, Switzerland). With that aim, volumes of interest (VOIs) were manually drawn in the lungs, liver, bladder, stomach, kidneys, spleen, tumour, heart and thyroid gland using the CT images as anatomical reference. VOIs were then transferred to the PET images and time activity curves (decay corrected) were obtained for each organ as cps/cm^3^. Curves were transformed into real activity (Bq/cm^3^) curves. Injected dose normalization was finally applied to data to get time activity curves as percentage of injected dose per cm^3^ of tissue.

### 3.7. Ex Vivo Studies

Ion beam microscopy (IBM) studies were performed at the LIPSION nanoprobe at Leipzig University using a 2.25 MeV proton beam with a spot size of approximately 1 µm and supplied by Singletron™ particle accelerator (HVEE, Amersfoort, NL). Under vacuum of 10^−6^ Torr two ion beam microscope techniques, such as micro-proton induced X-ray emission (µPIXE) and micro-Rutherford backscattering (µRBS), were used simultaneously to study the spatial distribution of elements originated from tissue and NPs. Extracted µRBS spectra from the region of interest were analyzed by using SIMNRA 6.06 software (Dr. Matej Mayer, MPI of plasmaphysic, Garching, GE) to determine accumulated charge, area density (atoms/cm^2^) and element matrix composition (C, N, O). These parameters were used as input for µPIXE analysis by means of GeoPIXE 5.1 software (CSIRO Earth Science and Resource Engineering, Clayton, Australia) to quantify element concentration in the tissue of NP treated mice. The detailed procedure is described elsewhere [61,77].

Confocal Raman microspectroscopy (CRM) analyses were performed using an Alpha300 R microscope (WITec GmbH, Ulm, Germany) equipped with a 532 nm laser source, a 600 g mm^−1^ grating and a charge-coupled device (CCD) cooled down to −61 °C. All measurements were conducted using a 63x water immersion objective (W Plan-Apochromat 63x/1.0, Zeiss, Oberkochen, Germany). Raman spectra were collected pixel-wise in x-y plane with an integration time of about 70 µs. Acquired spectra were processed using the Project FOUR PLUS 4.0 software (WITec GmbH, Ulm, Germany).

## 4. Conclusions

Size and shape-tuned gold NPs (AuNPs) stabilised with polyethylene glycol (PEG) and functionalized with the boron-rich anion cobalt *bis*(dicarbollide) have been prepared and characterised. The incorporation of the positron emitter ^124^I either at the core or at the shell of the NPs enabled the determination of the in vivo biodistribution in a xenograft mouse model of human fibroblastoma. The NPs showed good in vivo stability but poor accumulation in the tumour. These results suggest that appropriate modification of the gold core in terms of size and shape is required in order to develop promising BNCT drug candidates. 

## Supplementary Materials

The following are available online, Figure S1: ^1^H-NMR of [**3**]^−^, Figure S2: ^11^B-NMR of [**3**]^−^; Figure S3: ^13^C-NMR of [**3**]^−^; Figure S4: ^1^H-NMR of [**4**]^−^; Figure S5: ^11^B-NMR of [**4**]^−^; Figure S6: ^13^C-NMR of [**4**]^−^; Figure S7: ^1^H-NMR of [**5**]^−^; Figure S8: ^11^B-NMR of [**5**]^−^; Figure S9: ^13^C-NMR of [**5**]^−^; Figure S10: ^1^H-NMR of [**6**]^−^; Figure S11: ^11^B-NMR of [**6**]^−^; Figure S12: ^13^C-NMR of [**6**]^−^.
