# Supplementary material for: Gold Nanoparticles as Boron Carriers for Boron Neutron Capture Therapy: Synthesis, Radiolabelling and In Vivo Evaluation
Source: Molecules. 2019 Oct 7;24(19):3609. doi: 10.3390/molecules24193609 (PMC6804187; doi:10.3390/molecules24193609)
Supplement: Supplementary file 1 [file molecules-24-03609-s001.pdf]

Supplementary information

Gold nanoparticles as boron carriers for boron neutron capture therapy: synthesis, radiolabelling and *in vivo* evaluation.

Krishna R. Pulagam [1], Kiran B. Gona [1,2], Vanessa Gómez-Vallejo [3], Zuriñe Baz [1], Unai Cossío [4] and Jordi Llop [1,5,*]

[1] Radiochemistry and Nuclear Imaging Group, CIC biomaGUNE, San Sebastian, Spain; krpulagam@cicbiomagune.es (K. R. P.); zbaz@cicbiomagune.es (Z. B.)
[2] Current affiliations: (a) Cardiovascular Molecular Imaging Laboratory, Section of Cardiovascular Medicine and Yale Cardiovascular Research Center, Yale University School of Medicine, New Haven, CT (USA); (b) Veterans Affairs Connecticut Healthcare System, West Haven, CT (USA).
[3] Radiochemistry Platform, CIC biomaGUNE, San Sebastian, Spain; vgomez@cicbiomagune.es
[4] Radioimaging and Image Analysis Platform, CIC biomaGUNE, San Sebastian, Spain; ucossio@cicbiomagune.es
[5] Centro de Investigación Biomédica en red Enfermedades Respiratorias - CIBERES
* Correspondence: jllop@cicbiomagune.es; Tel.: +34-943-005-333 (J.L.)

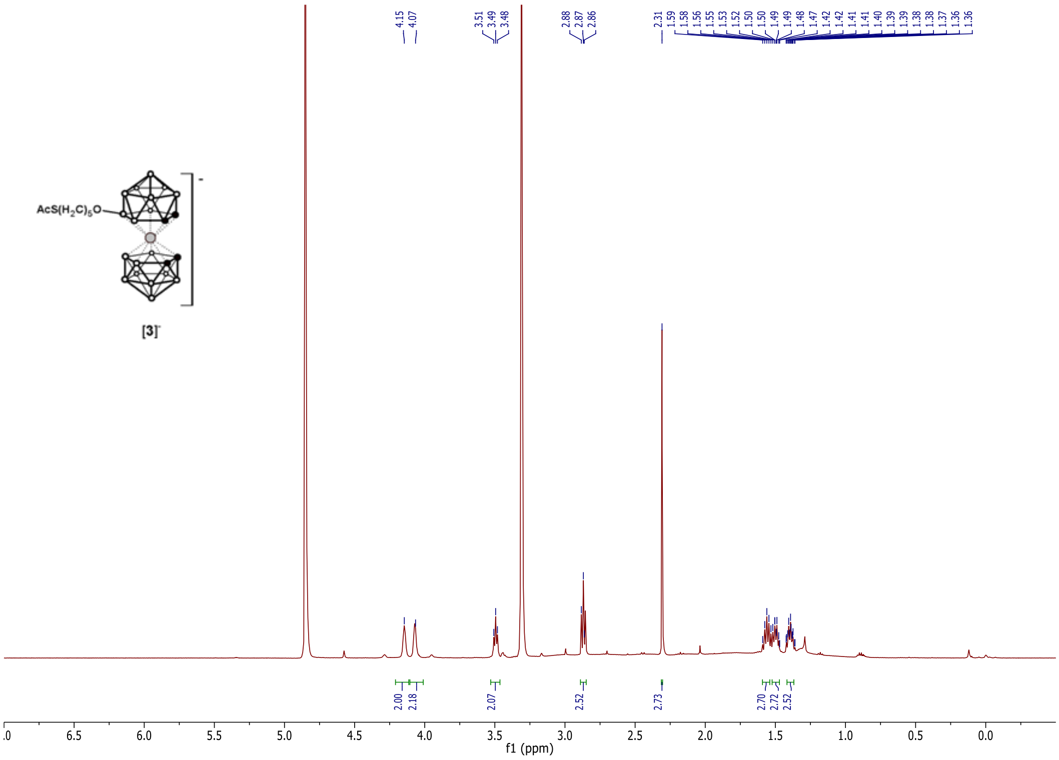

Figure S1. 500 MHz ^{1}H NMR spectrum of **[3]**$^{-}$ in methanol-d_4

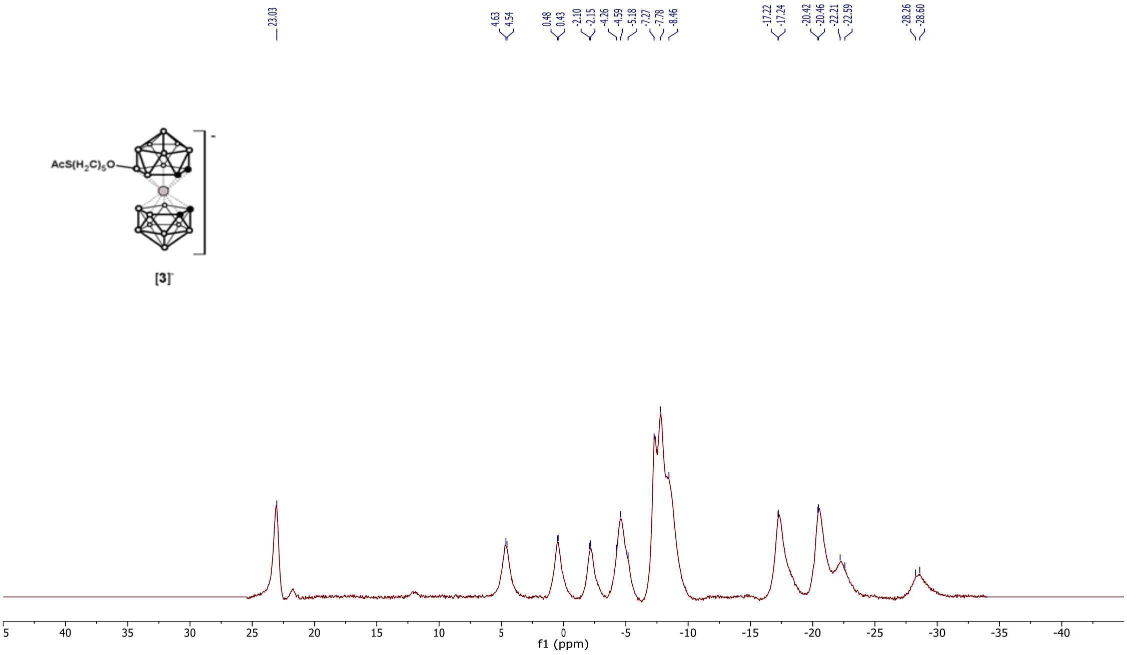

Figure S2. 160 MHz ^{11}B NMR spectrum of **[3]**$^{-}$ in methanol-d_4

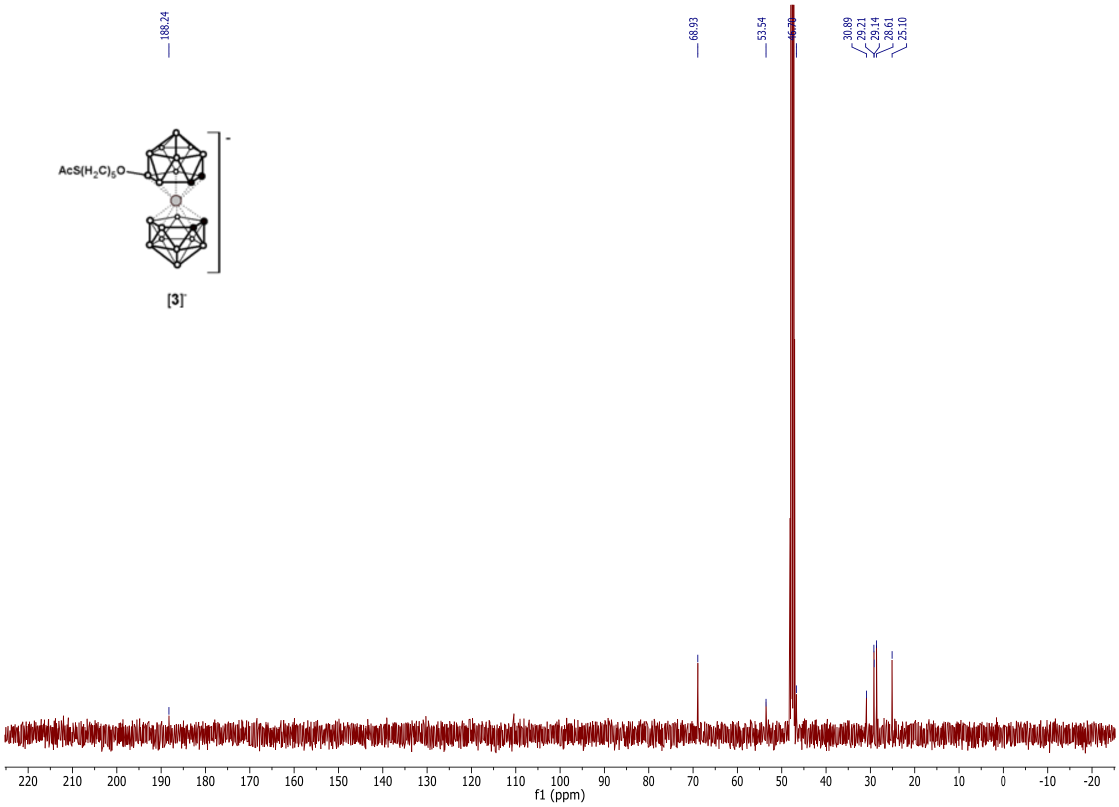

Figure S3. 126 MHz ^{13}C NMR spectrum of **[3]**$^-$ in methanol-d_4

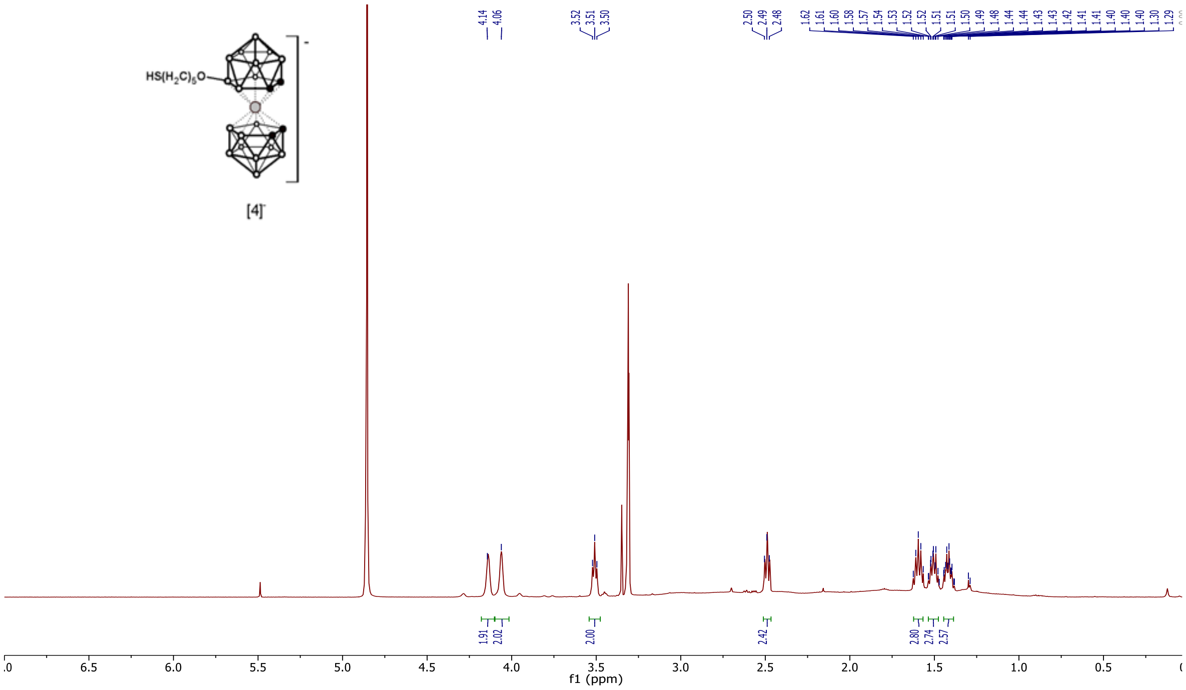

Figure S4. 500 MHz ^1H NMR spectrum of **[4]**$^-$ in methanol-d_4

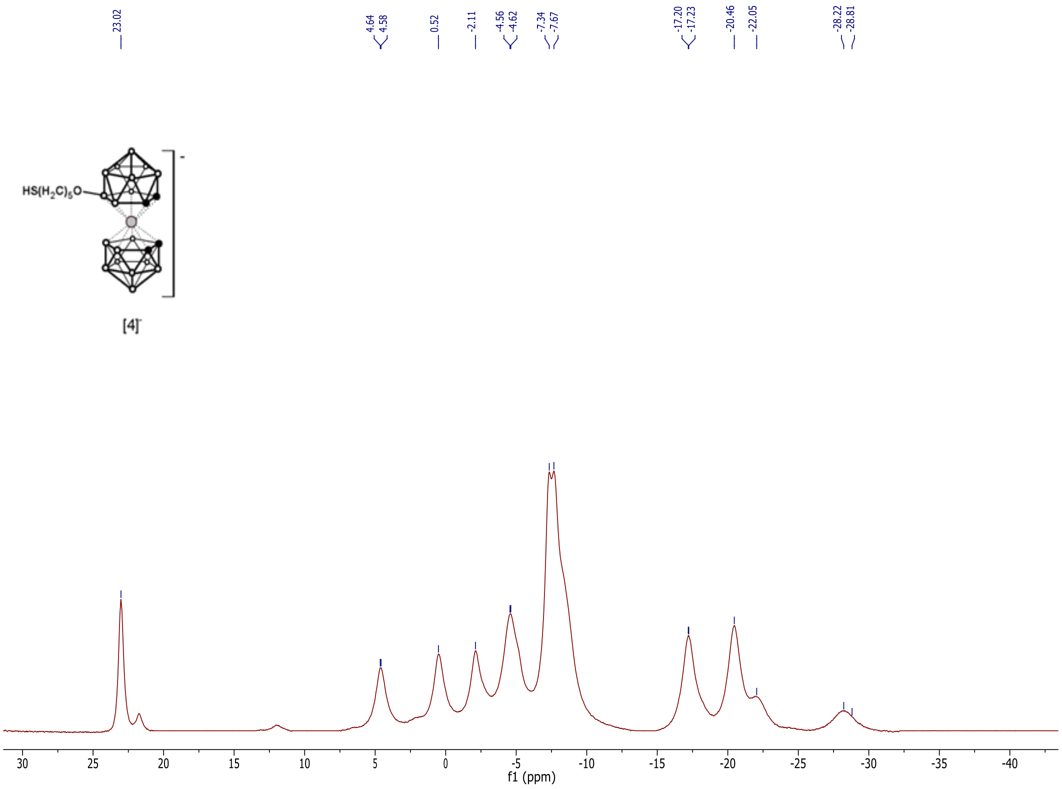

Figure S5. 160 MHz ^{11}B NMR spectrum of **[4]**$^{-}$ in methanol-d_4

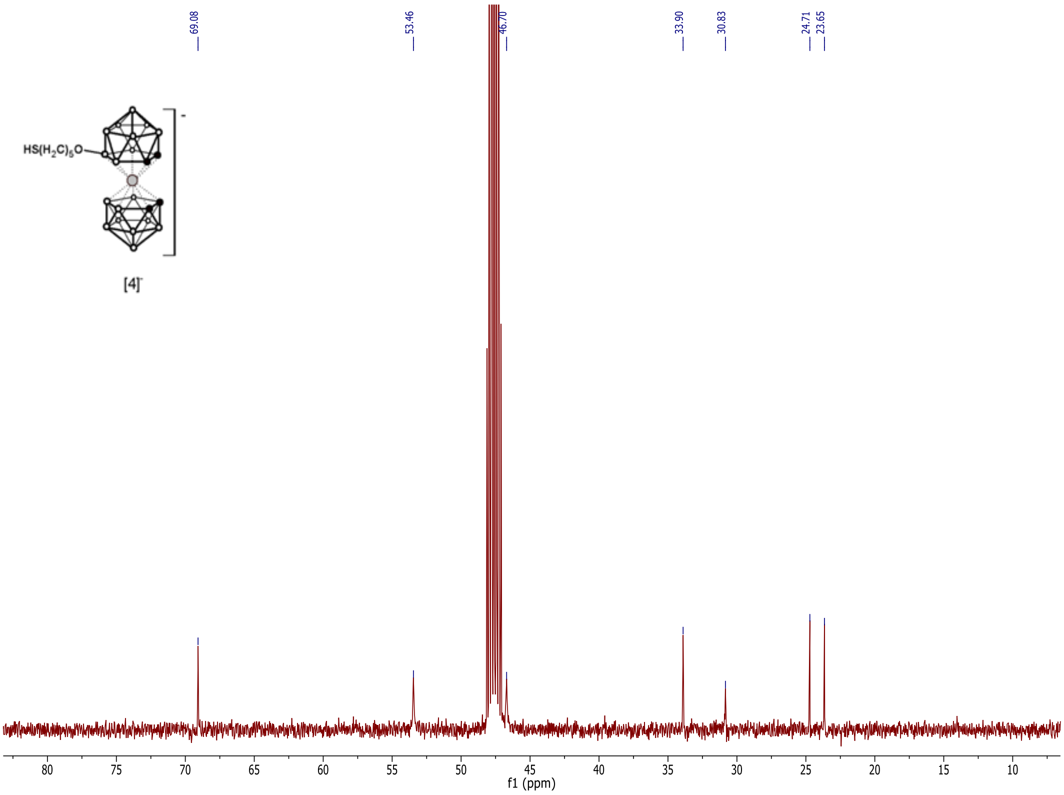

Figure S6. 126 MHz ^{13}C NMR spectrum of **[4]**$^{-}$ in methanol-d_4

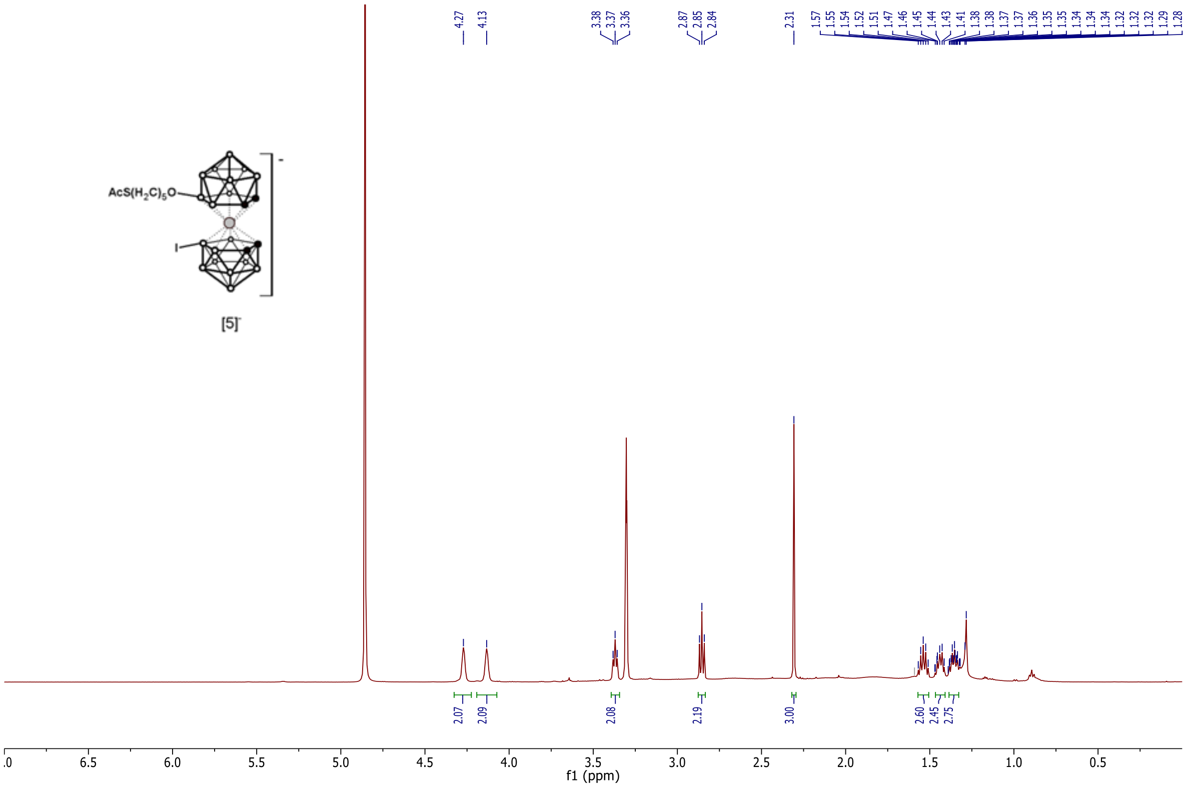

Figure S7. 500 MHz ^{1}H NMR spectrum of **[5]**$^{-}$ in methanol-d$_4$

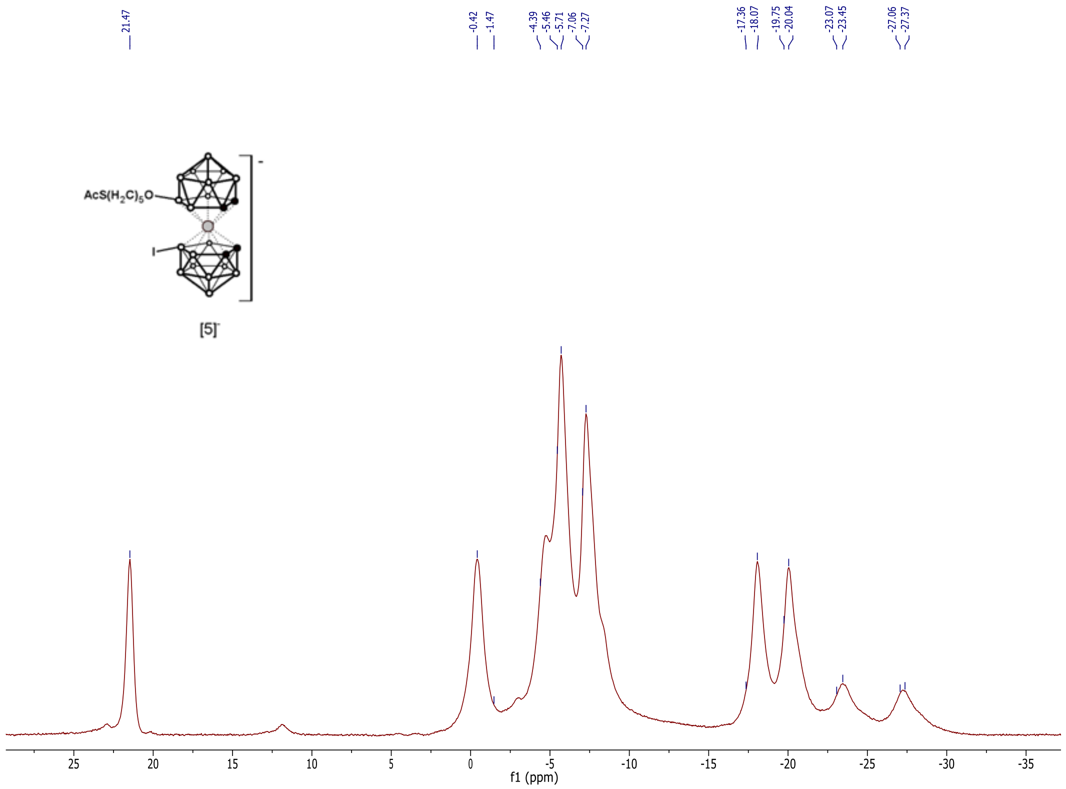

Figure S8. 160 MHz ^{11}B NMR spectrum of **[5]**$^{-}$ in methanol-d$_4$

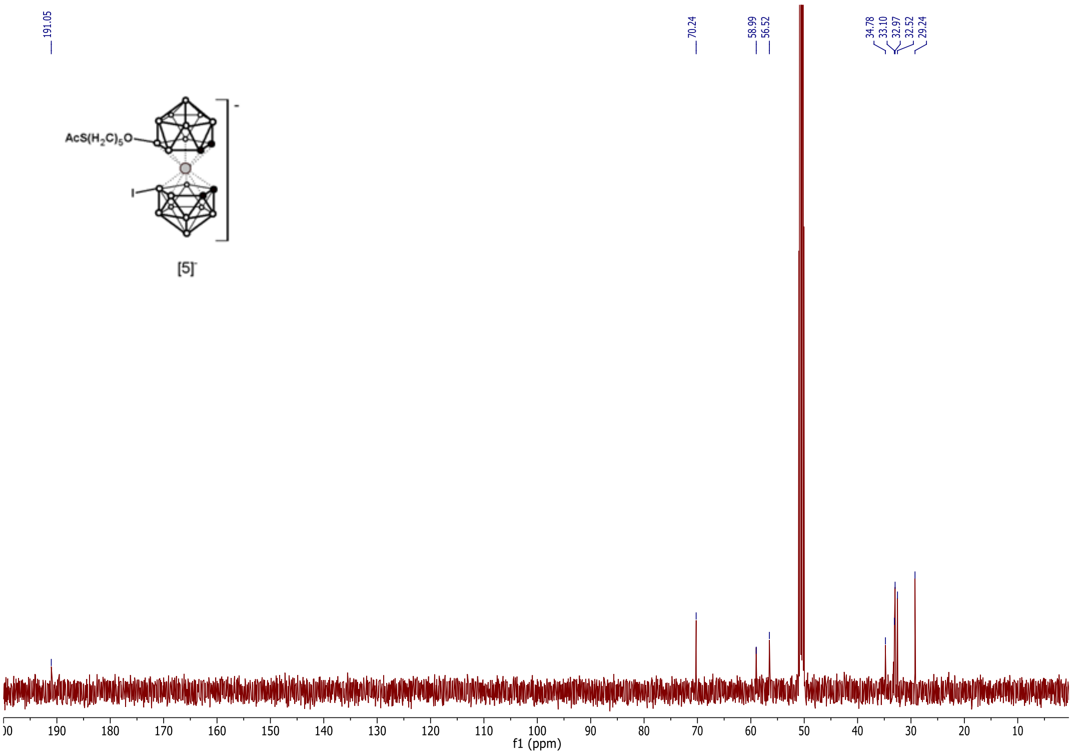

Figure S9. 126 MHz ^{13}C NMR spectrum of **[5]**$^-$ in methanol-d$_4$

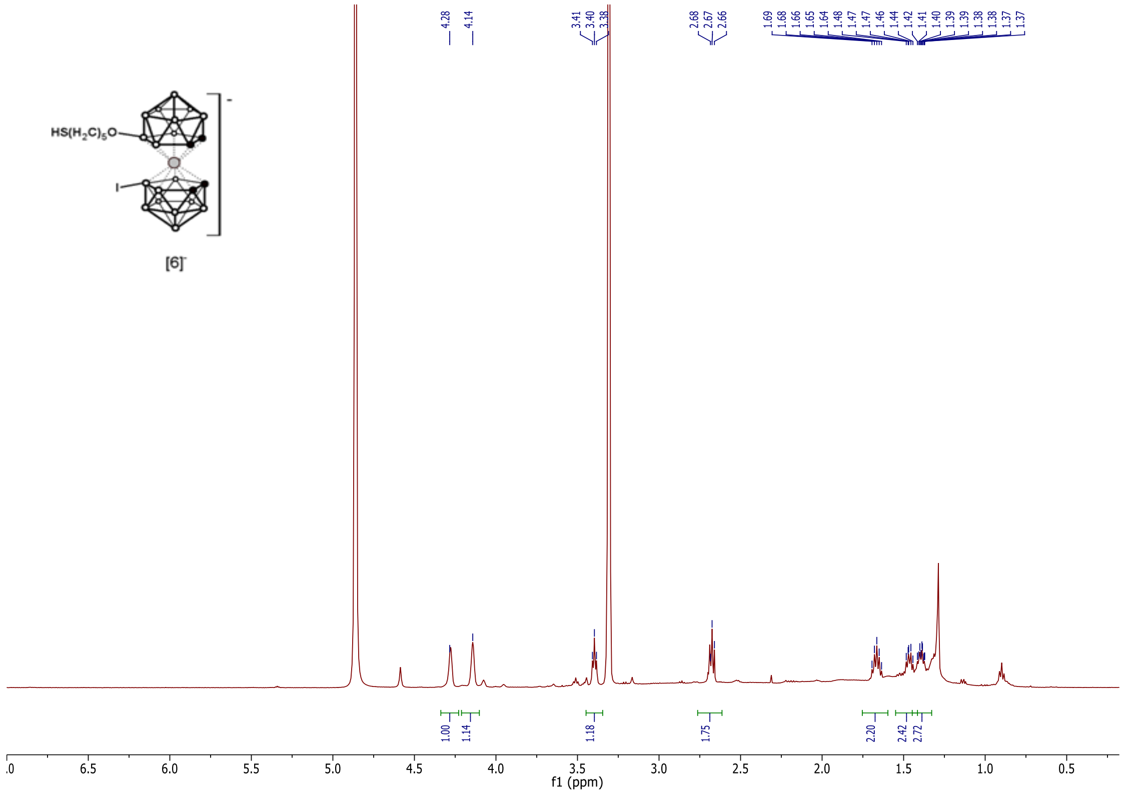

Figure S10. 500 MHz ^{1}H NMR spectrum of **[6]**$^-$ in methanol-d$_4$

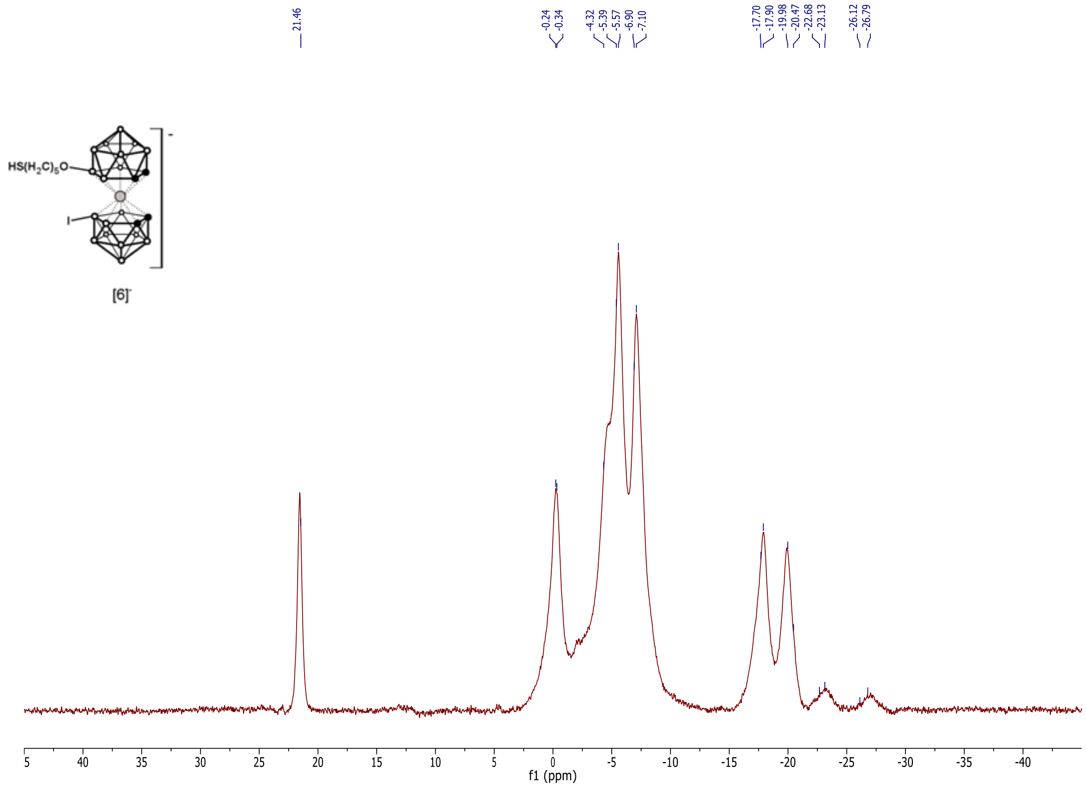

Figure S11. 160 MHz ^{11}B NMR spectrum of **[6]**$^-$ in methanol-d$_4$

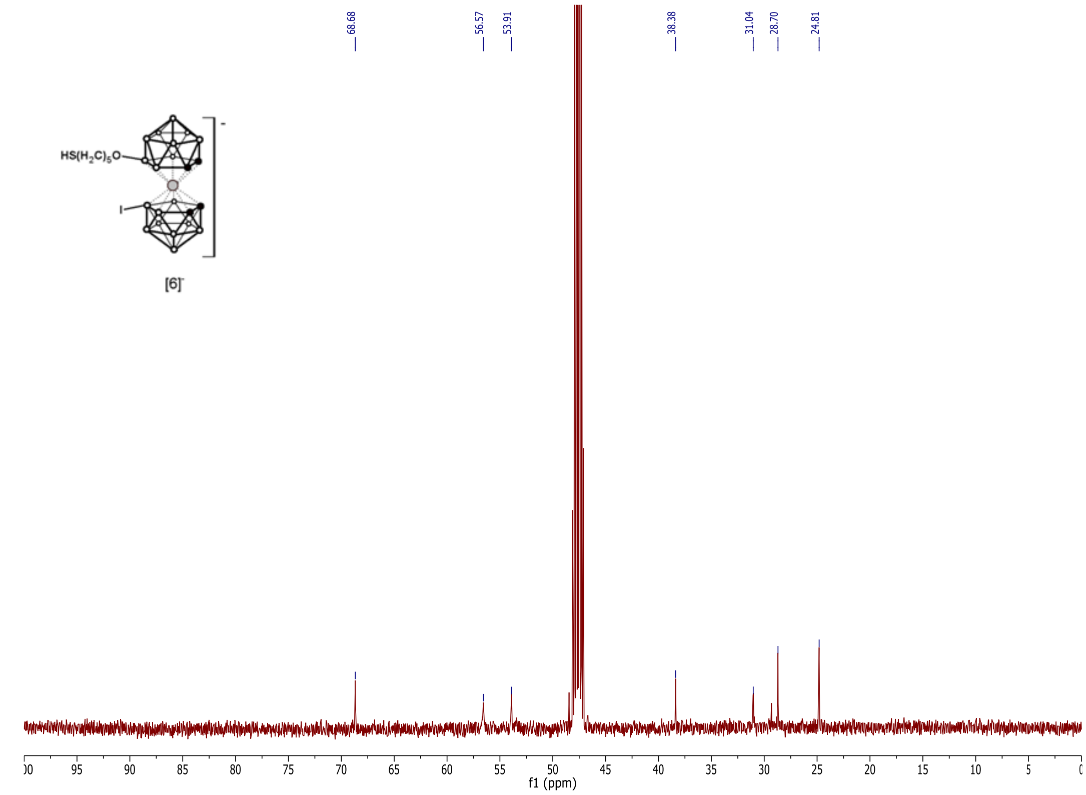

Figure S12. 126 MHz ^{13}C NMR spectrum of **[6]**$^-$ in methanol-d$_4$
